# A Case Series of Emphysematous Pyelonephritis

**DOI:** 10.1155/2014/587926

**Published:** 2014-04-09

**Authors:** Camelia Arsene, Abhijit Saste, Shankar Arul, Janee Mestrovich, Revark Kammo, Mohammed Elbashir, Gregory Berger

**Affiliations:** ^1^Department of Medicine, Sinai-Grace Hospital, Detroit Medical Center/Wayne State School of Medicine, 4th Floor, 6071 West Outer Drive, Detroit, MI 48235, USA; ^2^Department of Emergency Medicine, Detroit Receiving Hospital/Detroit Medical Center, 4201 Street Antoine, Suite 3R, Detroit, MI 48201, USA

## Abstract

*Introduction*. Emphysematous pyelonephritis (EPN) is an uncommon infection characterized by gas in the renal parenchyma and surrounding tissues. It is rapidly progressive, requiring appropriate therapy to salvage the infected kidney. *Case Description*. The case series presents 5 patients with a clinical and radiologic diagnosis of EPN. Each patient had a unique predisposing factor for developing EPN. Early goal directed therapy with intravenous fluids and antibiotics was given. This was followed by less invasive urologic interventions in an attempt to avoid nephrectomy and thereby salvage the infected kidney. All five patients were discharged in clinically stable conditions. *Discussion and Conclusion*. This case series provides added practice based support to available literature for managing EPN. Early goal directed medical therapy for sepsis coupled with interventional urologic procedures is a valuable alternative to circumvent an upfront emergent nephrectomy, except in cases where a fulminant infection may be present at the time of admission or develop later on in the course of the patients illness despite conservative line of therapy. It also highlights the importance of considering a diagnosis of EPN in patients with urinary infections, who have certain common predisposing factors listed in our case series.

## 1. Introduction

Emphysematous pyelonephritis (EPN) is a rare clinical condition characterized by the presence of gas in the renal system, most often in the parenchyma, but also extending to surrounding perinephric tissues. It is caused by gas-forming organisms, most commonly* Escherichia coli (E. coli),* in addition to* Klebsiella, Clostridium, Candida, Aspergillus, Cryptococcus, *and* Amoeba* [1].

Shultz and Klorfein originally described the clinical entity in 1962, although there is evidence that the medical field had knowledge of this entity in the late 1800 [2]. Although the clinical presentation of EPN is similar to uncomplicated pyelonephritis, it is a much more aggressive disease with high morbidity and mortality with estimates as high as 90% mortality [3]. Huang and Tseng have described a large case series involving 48 patients with EPN which showed that with different treatment modalities the overall mortality was 18.8% [2].

The pathogenesis of this disease is thought to involve many different predisposing factors including high tissue glucose concentrations, presence of gas-forming organisms, impaired vascular supply, impaired immune system, and ureteral obstruction [4]. It is still unknown why some people develop EPN while many others simply develop a conventional urinary tract infection.

The aim of this case series is to add to the current literature by providing information regarding our experience in diagnosing and successfully treating five patients diagnosed with EPN at our institution.

## 2. Case Series Description

From the year 1999 to 2009 a total of 2086 medical records at our institution were reviewed, looking particularly at the International Classification of Diseases (ICD) version 9 code of 590.0. The ICD codes for operations on the kidney were also reviewed, in particular code 55.0.

Patients were included in this study if they presented with symptoms and signs of upper urinary tract infection, fever with a positive urine culture, or pyuria without other identified infectious foci, with radiological evidence by computed tomography (CT) scan of gas accumulation in the collecting system, renal parenchyma, perinephric, or pararenal space, and were older than 18. The details of laboratory data and antibiotic therapy for each of the following cases are summarized in Table 1.

### 2.1. Case 1

**Figure 1:**
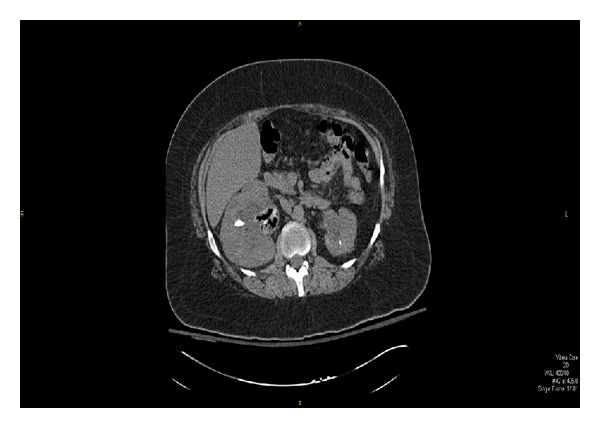
CT scan of the abdomen and pelvis showing marked right hydronephrosis with calculi, perinephric fat stranding, and gas in the renal pelvis.

A 39-year-old female with a past medical history of diabetes, hypertension, and renal calculi (with previous lithotripsy) presented with painful urination and dizziness of 3-day duration. She had stopped taking her insulin due to a lack of oral intake resulting from nausea and vomiting. At the time of admission the patient began to complain of right flank pain and subjective fevers. Laboratory data were as indicated in Table 1. CT scan showed marked right hydronephrosis with calculi, perinephric fat stranding, and gas in the renal pelvis (Figure 1). The patient was started empirically on intravenous (IV) Ciprofloxacin on arrival. This was then switched to (IV) Nafcillin (Table 1) as blood cultures collected on two separate days grew* Staphylococcus epidermidis* in both aerobic and anaerobic bottles. The* Staphylococcus epidermidis* was later found to be resistant to Oxacillin. The patient was therefore deescalated to targeted therapy on IV Vancomycin based on blood antibiotic susceptibility results (Table 1) and aggressively hydrated. Her initial urine cultures showed a mixed flora but repeat urine cultures showed no growth. A percutaneous nephrostomy catheter was placed on admission day 2. The patient continued to spike fevers. A ureteral stent was subsequently placed on admission day 6 along with a lithotripsy after which the percutaneous nephrostomy tube was removed. The patient was also successfully treated for her hyperosmolar nonketotic state. A follow-up technetium dimercaptosuccinic acid (DMSA) renogram done on admission day 7 showed prominent nonobstructive calyces especially on the right side and asymmetric renal function with the differential being 33% on the left and 67% on the right. The patient was subsequently discharged home in a stable condition and advised to follow up with both urology and infectious diseases specialists.

### 2.2. Case 2

**Figure 2:**
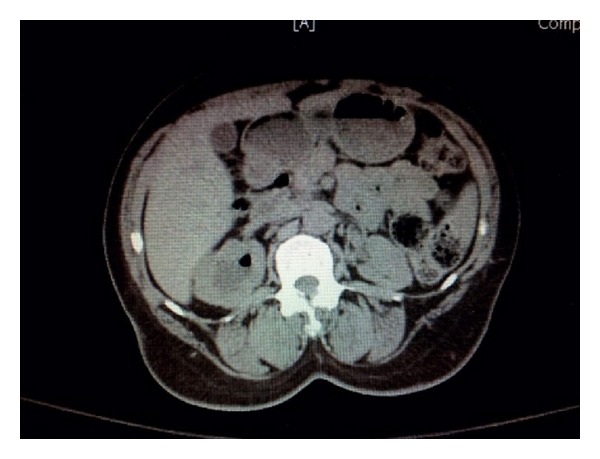
CT scan of the abdomen and pelvis showing right-sided ureteropelvic junction stenosis with moderate to marked hydronephrosis, air in the collecting system and intraparenchymal area, and increased echogenicity of the right renal parenchyma suggestive of parenchymal disease.

A 49-year-old female with past medical history of hypertension and IV drug abuse presented to the hospital with complaints of hemoptysis. On physical examination she was found to have right-sided flank pain and acute renal failure. Her laboratory data are presented in Table 1. A CT of the abdomen and pelvis showed right-sided ureteropelvic junction stenosis with moderate to marked hydronephrosis, air in the collecting system and intraparenchymal area, and increased echogenicity of the right renal parenchyma suggestive of parenchymal disease (Figure 2). The patient was immediately taken to the operating room for a ureteral stent placement. Here she was noted to have pus and air in the collecting system. Her urine cultures taken on admission grew* E. coli*. Her blood cultures were negative. The patient had been started on empiric IV Cefepime followed by IV Ceftriaxone based on antibiotic susceptibility results (Table 1). The patient did not have any improvement in her serum creatinine levels at the time of discharge. This was thought to be secondary to chronic kidney disease. After 4 days of treatment in the hospital, the patient was discharged home on oral Ciprofloxacin for 10 days based on urine susceptibility results to follow up with urology as an outpatient.

### 2.3. Case 3

**Figure 3:**
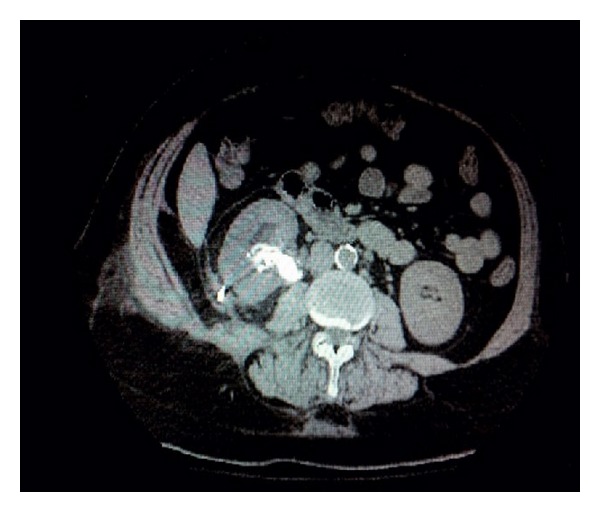
CT scan of the abdomen and pelvis showing a right staghorn calculus, right hydronephrosis with air in the collecting system, and perinephric stranding.

A 58-year-old female presented to the urology clinic with a repeat urinary tract infections. She also had intermittent nausea, vomiting, fevers, and chills. Her laboratory data are presented in Table 1. A CT scan of the abdomen showed a right staghorn calculus, right hydronephrosis with air in the collecting system, and perinephric stranding (Figure 3). Early goal directed therapy with fluids and antibiotics was initiated. The patient was sent to the hospital for a direct admission where she had an emergent nephrostomy tube placed. Her nephrostomy tube drained a mix of bloody and cloudy urine. The urine culture from nephrostomy grew* E. coli* and blood culture was negative. The patient was started on empiric IV Ciprofloxacin followed by oral Bactrim DS for 30 days (Table 1) or until a percutaneous nephrolithotomy was completed as an outpatient. She was discharged home on admission day 3 in a clinically improved condition.

### 2.4. Case 4

**Figure 4:**
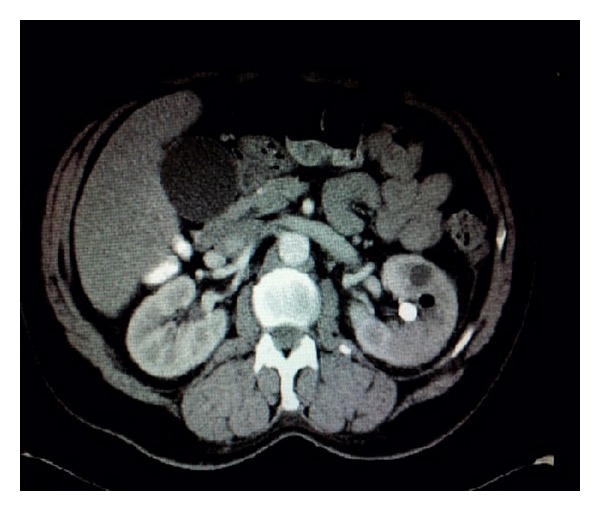
CT scan of the abdomen and pelvis showing left renal calculi with foci or air in the renal parenchyma.

A 61-year-old male with a past medical history of hypertension, gastroesophageal reflux disease, and drug abuse was brought to the emergency room with 3 days of abdominal pain and fever. The patient had epigastric pain along with nausea and vomiting. On clinical examination the patient was noted to be afebrile but did have bilateral costovertebral angle tenderness with the left side being worse than the right. Her laboratory data are presented in Table 1. A CT scan of the abdomen showed left renal calculi with foci or air in the renal parenchyma (Figure 4). Early goal directed therapy with fluids and antibiotics was initiated. Blood cultures and urine cultures grew* E. coli*. The patient was initially started on empiric IV Zosyn and IV Ceftriaxone followed by IV Ciprofloxacin and IV Ceftriaxone based on susceptibility results (Table 1). The patient became febrile during his stay and a repeat CT of the abdomen showed an abscess of the left kidney. Therefore, on admission day 9 the patient underwent a nephrectomy. Interventional radiology was of the opinion that a nephrostomy tube would be technically challenging to place in this patient and therefore was not undertaken. After the nephrectomy the patient showed marked clinical improvement. He was discharged home 4 days later in a clinically stable condition on a course of IV Ciprofloxacin and IV Ceftriaxone for 14 days and advised to follow up with urology and infectious diseases.

### 2.5. Case 5

**Figure 5:**
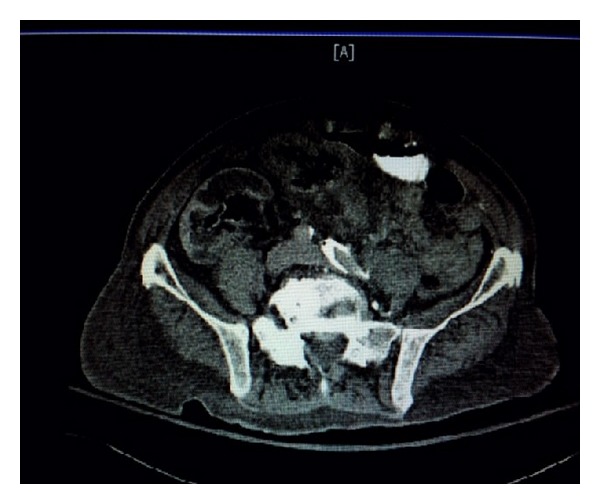
CT scan of the abdomen and pelvis showing an edematous transplanted kidney with air in the intraparenchymal and pelvic regions and the bladder.

A 65-year-old male with a past medical history of end stage renal disease status after kidney transplant, atrial fibrillation, and hypertension presented to the emergency room with complaints of abdominal pain for one week. He was known to have a failed kidney transplant at the time of admission as per his outpatient visit records. The suspicion for an infection or infarction of the kidney graft was high based on the patient being on immunosuppressant medications and having atrial fibrillation, respectively. His laboratory data are presented in Table 1. No urine was available for analysis as the patient was a hemodialysis patient. Blood cultures were negative. A CT of the abdomen and pelvis showed an edematous transplanted kidney with air in the intraparenchymal region and the urinary bladder (Figure 5). The patient also had a small bowel obstruction. Early goal directed therapy with fluids and antibiotics was started. He was started on empiric IV Unasyn (Table 1). The concern for a more fulminant EPN was high and the patient was taken to the operating room for an emergent transplant nephrectomy and hemicolectomy with small bowel resection. Postoperatively the patient developed acute respiratory failure secondary to* Acinetobacter* pneumonia and had to be admitted to the intensive care unit (ICU) service. He was started on IV Imipenem to which he responded with resolution of his pneumonia. He developed an ileus and required a course of total parenteral nutrition. In the ICU the patient developed* Clostridium difficile* colitis. He was successfully treated for* Clostridium difficile* colitis with oral Vancomycin. He made complete clinical recovery and was transferred to the medical floor where his clinical course was eventful for resolution of cough, dyspnea, fever, diarrhea, and abdominal pain. He was discharged on a course of IV Imipenem and IV Unasyn to complete a total of 14 days.

## 3. Discussion

EPN is a severe, necrotizing infection characterized by bacterial production of gas within the renal parenchyma. The conditions required for the generation of EPN are

1. the presence of pathogenic bacteria capable of mixed acid fermentation [5],
2. high levels of glucose in tissue,
3. impaired tissue perfusion [6].

These factors can work collectively resulting in a rapid progression of the disease; therefore, the level of suspicion should increase in conjunction with number of predisposing conditions. For example, local tissue ischemia in the presence of gas-forming bacteria will exacerbate tissue destruction, encourage purulent infection, and inhibit the removal of locally produced gas [5].

EPN in renal transplant recipients is a rare condition, with only 22 such cases reported in literature reviews [4, 7]. Though it is rare, it carries the devastating possibility of graft loss and is associated with high mortality. It should therefore be considered as a differential diagnosis in transplant patients presenting with signs and symptoms of pyelonephritis. Renal transplant recipients are treated with extended courses of immunosuppressive medications that create a milieu favorable to urinary tract infections [2, 7]. In addition, diabetes is currently the leading cause of end stage renal disease. Therefore, most renal transplant recipients have coexisting diabetes, thereby amplifying the potential for developing EPN [6, 8, 9]. We had one such case in our review series. True to form, the patient presented with symptoms suggestive of pyelonephritis. He did have EPN and a small bowel obstruction. He was managed with a transplant nephrectomy, as the patient was hemodynamically unstable and had a very fulminant picture on imaging studies with gas not only in the parenchyma of the kidneys but also extending into the pelvic regions and tracking along the urinary bladder. The patient was predisposed to a more clinically aggressive infection because of being on immunosuppressive therapy. The patient's compromised immune system rendered him unable to fight off infection from these gas-forming bacteria that result in EPN. However, if immunosuppressive drug therapy was the only risk factor in increasing his likelihood for the development of fulminant EPN, the prevalence of EPN in transplant patients would be higher. Another contributor to his presentation could be the presence of atrial fibrillation and/or hypertension, both of which increase the likelihood of an ischemic insult favoring the propagation of virulent gas producing bacteria.

The pathogenesis of gas formation requires pathogenic bacteria capable of mixed acid fermentation, a hyperglycemic environment, and localized tissue ischemia. Because a hyperglycemic environment is one of the requirements in gas formation, it only makes sense that diabetes is a significant predisposing factor. It has been estimated that up to 95% of EPN cases have underlying uncontrolled diabetes mellitus [10]. Furthermore, hyperglycemia in association with impaired blood supply to the kidneys from vasculopathy—both of which are prevalent in diabetic patients—facilitates the process of anaerobic metabolism [11]. Although diabetes is the number one predisposing factor for EPN development, we only had one patient with diabetes. The remainder of patients had predisposing factors such as ureteric obstruction or immunological impairment [6].

Our case review presents three patients with renal calculi. Risk of developing EPN in patients with urinary tract obstruction is about 25–40%, with ureteral obstruction being the second most common predisposing factor in those diagnosed with EPN [10, 12]. Current evidence suggests females are more susceptible to EPN because they are also more susceptible to urinary tract infections [2, 7, 13]. Ureteral obstruction causes local tissue ischemia which can provoke an infection in a number of ways such as exacerbating local tissue destruction, encouraging purulent infection, and inhibiting the removal of locally produced gas [2, 5, 13]. Multiple reasons exist, as a result of which obstructive calculi increase the likelihood of infection. One possibility is the stone providing a nidus for infection from where the disease could spread further on. Another possibility is that the obstructing calculus causes stagnation/reflux of urine and therefore a lack of laminar flow in the ureteral system. This would make it easier for the pathogens to ascend proximally up the urethra and ureters resulting in infection.

In our case series, four out of the five patients had hypertension, and the medical records did not state if the fifth patient had hypertension. As mentioned earlier, ischemia is a known predisposing factor for EPN. Hypertension causes ischemia through mechanisms such as arteriosclerosis and glomerulosclerosis.

*E. coli* is noted to be a very common pathogen in EPN [1, 2, 4, 7, 13, 14]. Our case series had three patients with* E. coli* positive urine cultures. One patient had a blood culture that grew* Staphylococcus epidermidis*. One of the patients did not have urine cultures recorded in the medical records as he was a dialysis patient.

The clinical approach to treating patients with EPN has changed over the years. Due to advances in medical imaging, interventional radiology, newer more effective antibiotic therapy, and readily available intensive care integrated with dialytic support, patients with EPN have much better outcomes. Managing EPN more conservatively has thus become the standard of care [15]. Our approach to patient management is in accordance with current evidence based protocols. All the patients were treated with broad-spectrum antibiotics, which were subsequently adjusted based on culture results. They received early goal directed therapy for their sepsis. Recent reviews of the management of EPN propose that percutaneous drainage should be part of the initial management strategy for EPN [16]. In patients with extensive/fulminant disease with hemodynamic compromise many have determined that, together with fluid resuscitation and antibiotics, immediate nephrectomy should not be delayed for the successful management of EPN [17–19]. Our case series seems to further advocate this management approach, as two patients had percutaneous nephrostomy tubes placed, three had ureteral stents placed, and two had to be taken in for emergency nephrectomies; none of our patients were solely medically managed.

Even though our number of cases was small, there were no mortalities reported. We attempted to explain this phenomenon by studying the current literature. One large review covering 10 studies on 210 patients with EPN found that the mortality from medical management alone was 50%, medical management combined with emergency nephrectomy was 25%, and medical management combined with percutaneous drainage was 13.5% [17]. Mortality was significantly less in patients undergoing percutaneous drainage compared to other treatments. Of the patients who underwent medical treatment with percutaneous drainage, a small number (15) underwent elective nephrectomy and mortality was 6.6% [17]. Percutaneous drainage should therefore be a part of the initial management strategy for EPN. This strategy is associated with a lower mortality than medical management or emergency nephrectomy. Delayed elective nephrectomy may be eventually required in some patients. The advantages of percutaneous drainage include increased patient stability for subsequent reversal of some of the underlying contributory factors. This in turn then decreases the risk of adverse events should a nephrectomy be eventually needed.

## 4. Conclusions

Our clinical experience and data suggest that early goal directed therapy with IV antibiotics and fluid resuscitation together with less invasive interventions such as nephrostomy tubes and ureteral stents can provide viable alternatives to nephrectomies in early stages of EPN, thereby attempting to salvage kidneys. Nephrectomies could be reserved for the more fulminant cases of EPN presenting with hemodynamic compromise or progressive infections despite percutaneous drainage and medical therapy. Additionally, our case series highlights the importance of considering EPN to be a clinical diagnosis in patients having the aforementioned predisposing risk factors.

## Figures and Tables

**Table 1:** Patient laboratory data and antibiotic treatment.

Case/data	Metabolic panel	Blood count	Urine analysis	Antibiotics
Case 1	Na^+^ 125 mEq/L K^+^ 6.6 mEq/L Cl^−^ 87 mEq/L HCO_3_^−^ 24 mEq/L BUN 23 mg/dL Cr 2.7 mg/dL Glu 917 mg/dL	WBC 12.5 k/mm^3^ Hgb 14 g/dL Hct 41% Plt 273 k/mm^3^	Turbid 4+ bacteria >100 WBC >100 RBC 3+ blood 2+ leukocyte esterase Nitrate negative	Empiric Nafcillin 1 g IV q6 followed by Vancomycin 1 g IV q12 for 14-day monotherapy based on susceptibility results

Case 2	Na^+^ 140 mEq/L K^+^ 4.2 mEq/L Cl^−^ 104 mEq/L HCO_3_^−^ 22 mEq/L BUN 32 mg/dL Cr 2.9 mg/dL Glu 98 mg/dL	WBC 16.8 k/mm^3^ Hgb 11.5 g/dL Hct 37.6% Plt 271 k/mm^3^	Turbid 1+ bacteria 20–50 WBC 0 RBC 1+ leukocyte esterase Nitrate positive	Empiric Cefepime 1 g IV q12 followed by Ceftriaxone 1 g IV q24 based on susceptibility results. Thereafter Ciprofloxacin 500 mg po q24 for 10 days based on susceptibility results

Case 3	Na^+^ 143 mEq/L K^+^ 3.6 mEq/L Cl^−^ 109 mEq/L HCO_3_^−^ 21 mEq/L BUN 21 mg/dL Cr 0.7 mg/dL Glu 98 mg/dL	WBC 17.5 k/mm^3^ Hgb 13.2 g/dL Hct 41.9% Plt 224 k/mm^3^	Turbid 4+ bacteria >100 WBC >100 RBC 3+ blood 3+ leukocyte esterase Nitrate negative	Empiric Ciprofloxacin 400 mg IV q12 followed by Bactrim DS po BID for 30 days based on susceptibility results

Case 4	Na^+^ 136 mEq/L K^+^ 4.7 mEq/L Cl^−^ 104 mEq/L HCO_3_^−^ 24 mEq/L BUN 12 mg/dL Cr 1.2 mg/dL Glu 105 mg/dL	WBC 18.8 k/mm^3^ Hgb 10.4 g/dL Hct 29.7% Plt 387 k/mm^3^	Turbid 24+ bacteria 10–20 WBC 5–10 RBC 1+ blood 3+ leukocyte esterase Nitrate positive	Empiric Zosyn 3.3.7 IV q6 and IV Ceftriaxone 2 gm q24 followed by Ciprofloxacin 400 mg IV q12 along with Ceftriaxone 2 gm IV q24 g for a total of 14 days based on susceptibility results

Case 5	Na^+^ 138 mEq/L K^+^ 4.6 mEq/L Cl^−^ 109 mEq/L HCO_3_^−^ 19 mEq/L BUN 65 mg/dL Cr 6.5 mg/dL Glu 72 mg/dL	WBC 17.3 k/mm^3^ Hgb 9.7 g/dL Hct 30.3% Plt 293 k/mm^3^	Not available as patient is a hemodialysis patient	Unasyn 3 g IV q24 and Imipenem 500 mg IV q6 for 14 days
